# Astragalus polysaccharide promotes latent HIV-1 reactivation via exosome-mediated modulation of the PI3K/AKT/NF-κB axis

**DOI:** 10.20517/evcna.2025.148

**Published:** 2026-01-29

**Authors:** Fang Xu, Shumin Luo, Pengpeng Lu, Yiyue Wang, Guannan Sun, Chuanyun Li, Weihua Li

**Affiliations:** ^1^Beijing Institute of Hepatology, Beijing You An Hospital, Capital Medical University, Beijing 100069, China.; ^2^Beijing Institute of Infectious Diseases of Integrated Traditional Chinese and Western Medicine, Beijing You An Hospital, Capital Medical University, Beijing 100069, China.; ^3^Beijing You An Hospital, Capital Medical University, Beijing 100069, China.

**Keywords:** Exosome, HIV-1 reservoir, astragalus polysaccharide, PI3K/AKT/NF-κB signaling pathway

## Abstract

**Aim:** Reactivating the human immunodeficiency virus type 1 (HIV-1) reservoir is one of the key avenues toward a functional cure for acquired immunodeficiency syndrome (AIDS). However, clinically effective and safe latency-reversing agents remain lacking. Exosomes play an important role in regulating latent HIV-1. This study elucidates the capacity of exosomes derived from Astragalus polysaccharide (APS)-primed HIV-1 reservoir cells, J-Lat Full Length 10.6 (hereafter abbreviated as J-Lat 10.6), termed APS-Lat-EXO, to reactivate latent HIV-1 and investigates the underlying mechanisms.

**Methods:** J-Lat 10.6 cells were used as a model of HIV-1 latency. Cells were exposed to APS, and exosomes derived from APS-treated cells (APS-Lat-EXO) and untreated cells (Lat-EXO) were isolated. These exosomes were subsequently applied to J-Lat 10.6 cells. Latency reactivation was evaluated by flow cytometry, Enzyme-Linked ImmunoSorbent Assay (ELISA), and real-time quantitative polymerase chain reaction (RT-qPCR), which measured green fluorescent protein (GFP⁺) cell proportions, p24 (the HIV-1 capsid protein) levels, and group-specific antigen (Gag)/long terminal repeat (LTR) expression, respectively. Proteomic and microRNAs (miRNAs) analyses were performed to compare APS-Lat-EXO and Lat-EXO, and Western blot assays were used to assess activation of the phosphoinositide 3-kinase (PI3K)/protein kinase B (AKT)/nuclear factor kappa-light-chain-enhancer of activated B cells (NF-κB) signaling pathway.

**Results:** APS-Lat-EXO significantly reactivated latent HIV reservoirs in both J-Lat 10.6 cells and peripheral blood mononuclear cells from HIV-1-infected individuals, as indicated by increased GFP⁺ cell proportions, elevated p24 levels, and upregulated Gag/LTR expression. Proteomic and miRNA microarray analyses revealed notable differences between APS-Lat-EXO and Lat-EXO in protein profiles and miRNA composition. Functional enrichment analyses showed that differentially expressed proteins in APS-Lat-EXO were enriched in the PI3K/AKT pathway, while differentially expressed miRNAs were linked to NF-κB. *In vitro* experiments confirmed that APS-Lat-EXO reactivated latent HIV-1 via the PI3K/AKT/NF-κB pathway.

**Conclusion:** This study shows that APS-Lat-EXO is capable of inducing reactivation of latent HIV-1 through activation of the PI3K/AKT/NF-κB axis, highlighting its potential as a bio-derived latency-reversing candidate within the shock-and-kill framework.

## INTRODUCTION

Human immunodeficiency virus type 1 (HIV-1) remains a major threat to global public health. While combination antiretroviral therapy (ART) can durably control viral replication and transcription, slow clinical progression, and extend life expectancy, it fails to eliminate the virus due to persistent infection within the host. Once treatment is interrupted, HIV-1 often rebounds rapidly, leading to viral recrudescence and disease progression^[1,2]^. The fundamental reason is that HIV-1 establishes a long-term, stable latent infection in the host, forming the HIV-1 latent reservoir^[3,4]^. The latent reservoir is primarily maintained in quiescent memory CD4⁺ T lymphocytes, where the viral genome integrates into the host chromosome as a provirus without expressing viral proteins, thereby evading immune recognition and clearance^[5]^.

The existence of the HIV-1 latent reservoir is considered the greatest barrier to achieving a functional cure. One major strategy under investigation is “shock and kill”^[6,7]^, in which latency-reversing agents (LRAs) are used to reactivate silent proviruses, followed by ART, immune clearance, or other mechanisms to eliminate the reactivated infected cells and reduce the reservoir^[8,9]^. However, currently available LRAs, such as histone deacetylase inhibitors (HDACi) and protein kinase C (PKC) agonists, show limited efficacy and are often associated with cytotoxicity and nonspecific immune activation, which greatly restrict their clinical applicability^[10,11]^. Therefore, the development of novel LRAs with low toxicity and high efficacy has become an important direction in HIV-1 cure research.

In recent years, natural products and their derivatives have attracted attention as potential LRA candidates due to their favorable safety profiles and broad biological activities^[12,13]^. Astragalus polysaccharide (APS), the primary active component of the traditional Chinese medicine *Astragalus membranaceus*, exhibits diverse biological functions, including immunomodulatory, anti-inflammatory, and antiviral activities. In various disease models, APS has been shown to regulate cytokines, activate immune signaling pathways, and modulate cellular functions^[14-16]^. Accumulating studies suggest that pharmacological stimulation can alter the secretory profiles of cells, leading to the release of exosomes carrying diverse functional cargos, including proteins and microRNAs (miRNAs), which subsequently influence the biological activities of recipient cells^[17,18]^. As key vehicles for cell-to-cell communication, exosomes are deeply involved in the coordination of immune regulation, viral pathogenesis, and tumor-associated microenvironments^[19,20]^. Increasing evidence further indicates that exosomes contribute to the maintenance and disruption of HIV-1 latency. Notably, CD4⁺ T cells infected with HIV-1 have been reported to secrete exosomes enriched in viral-derived components, which can engage surrounding cells and facilitate the reactivation of latent HIV-1, ultimately triggering viral gene transcription^[21]^. This suggests that the regulatory effect of APS on immune signaling pathways may be amplified or targeted through exosomes as “cross-boundary messengers”, potentially serving as an important route for the reactivation of latent HIV-1. However, the relationship between APS and latent HIV-1 has not yet been reported.

Although previous studies have explored the application of cytokine-based LRAs and epigenetic drugs (such as HDACi and PKC agonists) in latent HIV reversal, these strategies are often associated with cytotoxicity and nonspecific immune activation, limiting their clinical applicability^[10,11]^. Furthermore, existing research on exosome-mediated HIV-1 activation has not focused on the activation efficiency of exosomes derived from cells treated with natural products^[22]^. In contrast, the exosomes derived from APS-primed HIV-1 reservoir cells, J-Lat Full Length 10.6 (J-Lat 10.6), hereafter referred to as APS-Lat-EXO, offer a novel and potentially safer approach.

In this study, we aimed to investigate the role of APS in latent HIV-1 infection by examining the effects of APS-treated cell-derived exosomes (APS-Lat-EXO) on latent HIV-1 reactivation and further elucidating the underlying mechanisms.

## METHODS

### Patients

Individuals living with HIV-1 and receiving ART were enrolled at Beijing You’an Hospital, Capital Medical University, China. Relevant clinical data, including plasma viral load and routine laboratory parameters, were extracted from the institutional electronic medical record system. All procedures adhered to the Declaration of Helsinki. The study protocol was approved by the Ethics Committee of Beijing You’an Hospital, Capital Medical University (LL-2024-075-K), and written informed consent was obtained from all participants prior to enrollment.

### Cell culture

The J-Lat Full Length 10.6 cell line was obtained from the National Institutes of Health (NIH) AIDS (acquired immunodeficiency syndrome) Reagent Program, USA. This Jurkat-derived model harbors an integrated full-length HIV-1 genome containing a green fluorescent protein (GFP) reporter, enabling quantification of viral reactivation upon latency reversal. Cells were plated at suitable densities in culture flasks, 6-well plates, or 24-well plates (Corning, USA) and cultured in RPMI 1640 medium (Cat. No. C11875500BT, Gibco, USA) supplemented with 10% fetal bovine serum (FBS; Cat. No. 10099141C, Gibco, USA). All cultures were maintained at 37 °C in a humidified incubator with 5% CO_2_.

### Exosome isolation

The J-Lat Full Length 10.6 cell line, a Jurkat-derived latency model harboring an integrated full-length HIV-1 genome with a GFP reporter, was employed for exosome production. APS powder (Cat. No. Z20040085, Tianjin Sino Pharmaceutical Co., Ltd., Tianjin, China; purity > 99.8%) was dissolved in phosphate-buffered saline (PBS, Cat. No. C10010500BT, Gibco, USA) and used to treat J-Lat 10.6 cells, while untreated cells were maintained as controls. Cells were cultured in RPMI 1640 medium (Cat. No. C11875500BT, Gibco, USA) supplemented with 10% exosome-depleted FBS (Cat. No. 10099141C, Gibco, USA). Following treatment, conditioned media from both groups were harvested and clarified by centrifugation at 10,000 × *g* for 30 min at 4 °C to remove cellular debris. Exosomes were subsequently isolated by ultracentrifugation using an Optima XPN-100 system (Beckman Coulter, USA) at 110,000 × *g* for 70 min at 4 °C. The resulting pellet was gently resuspended in PBS (Cat. No. C10010500BT, Gibco, USA; pH 7.4; without calcium chloride, magnesium chloride, or phenol red; osmolarity 280-315 milliosmoles per kilogram (mOsm/kg); sterilized by 0.22 μm filtration) and stored at 15-30 °C.

### Nanoparticle tracking analysis

Exosome samples were adjusted to final concentrations ranging from 1 × 10^7^ to 1 × 10^9^ particles/mL prior to analysis. Measurements were performed using a ZetaView PMX 110 instrument (Particle Metrix, Germany) equipped with a 405 nm laser. For each sample, 60 s videos were recorded at 30 frames per second. Vesicle size distribution and particle concentration were calculated from particle trajectories using ZetaView software (version 8.02.28).

### Transmission electron microscopy

For ultrastructural examination, 10 μL of exosome suspension was applied onto a copper grid and allowed to adsorb at room temperature for 1 min. Excess liquid was removed, and the grid was gently washed with sterile distilled water. Samples were then contrasted with uranyl acetate for 1 min and air-dried under an incandescent lamp for 2 min. Exosomes were subsequently examined and imaged using a transmission electron microscope (H-7650, Hitachi Ltd., Japan).

### Flow cytometry

Antibodies used for exosome detection included anti-CD9 [555371, BD Biosciences, San Jose, CA, USA; fluorescein isothiocyanate (FITC)], anti-CD63 [557305, BD Biosciences; phycoerythrin (PE)], and anti-CD81 [551112, BD Biosciences; allophycocyanin (APC)]. Following ultracentrifugation, exosomes were resuspended in PBS, incubated with the antibodies, and subjected to a second ultracentrifugation to remove unbound dyes. GFP expression in J-Lat 10.6 cells was simultaneously assessed. Data acquisition was performed using an Amnis imaging flow cytometer (Luminex, Austin, TX, USA) and a BD FACSCalibur system, with subsequent analysis conducted using IDEAS 6.2 software (Luminex) and FlowJo v10.6 (BD Biosciences). For exosome membrane labeling, PKH26 dye (MIDI26-1KT, Sigma-Aldrich, St. Louis, MO, USA; yellow fluorescence) was applied, and exosome binding was evaluated using the Amnis imaging flow cytometer.

### ELISA

HIV-1 p24, the viral capsid protein, was quantified in cell culture supernatants using a commercially available Enzyme-Linked Immunosorbent Assay (ELISA) kit (K12P24401, Beijing Keyue Zhongkai Biotechnology, China) according to the manufacturer’s instructions.

### RT-qPCR

Total RNA was isolated from J-Lat 10.6 cells and corresponding culture supernatants using the PureLink^TM^ RNA Micro Kit (12183016, Thermo Fisher Scientific, Waltham, MA, USA). Reverse transcription to generate complementary DNA (cDNA) was performed with the PrimeScript^TM^ 1st Strand cDNA Synthesis Kit (6110A, TaKaRa Bio Inc., Kusatsu, Shiga, Japan). Real-time quantitative polymerase chain reaction (PCR) (RT-qPCR) was conducted using the TB Green® Premix Ex Taq^TM^ (Tli RNaseH Plus) kit (RR420A, TaKaRa Bio Inc., Kusatsu, Shiga, Japan) on an ABI ViiA 7 Real-Time PCR System (Applied Biosystems, Foster City, CA, USA)^[23]^. Glyceraldehyde 3-phosphate dehydrogenase (GAPDH) served as the internal reference gene, and relative transcript levels were calculated using the 2^-ΔΔCt^ method. Primer sequences are listed in Supplementary Table 1.

### Western blotting

Exosomal proteins were extracted using radio-immunoprecipitation assay (RIPA) buffer (R0010, Sigma-Aldrich) supplemented with a protease inhibitor cocktail (P0100, Solarbio, Beijing, China)^[24]^. For cellular protein analysis, total proteins were prepared with RIPA lysis buffer (R0010, Sigma-Aldrich), while nuclear proteins were obtained using a nuclear/cytoplasmic protein extraction kit (78833, Thermo Fisher Scientific). Both phosphatase inhibitor cocktail (P1260, Solarbio) and protease inhibitor cocktail (P0100, Solarbio) were included in all lysis procedures. Protein lysates were clarified by centrifugation at 12,000 × *g* for 10 min, and the resulting supernatants were combined with sodium dodecyl sulfate-polyacrylamide gel electrophoresis (SDS-PAGE) sample loading buffer (P1040, Solarbio) followed by heat denaturation at 100 °C for 10 min. Equal amounts of protein were resolved on 10% SDS-PAGE gels and subsequently transferred onto polyvinylidene fluoride (PVDF) membranes (1620177, Bio-Rad, Hercules, CA, USA). Membranes were blocked with 5% non-fat milk and incubated overnight at 4 °C with primary antibodies against CD9 (sc-13118, Santa Cruz Biotechnology, Dallas, TX, USA), CD63 (sc-5275, Santa Cruz Biotechnology), CD81 (sc-166029, Santa Cruz Biotechnology), Calnexin (ab22595, Abcam, Cambridge, UK), β-actin (4970, Cell Signaling Technology, Danvers, MA, USA), phosphoinositide 3-kinase (PI3K, 4249, CST), phospho-PI3K (PA5-118549, Thermo Fisher Scientific), protein kinase B (AKT, 4691, CST), phospho-AKT (4060, CST), nuclear factor kappa-light-chain-enhancer of activated B cells (NF-κB, 8242, CST), phospho-NF-κB (3033, CST), and histone deacetylase 1 (HDAC1, 10197-1-AP, Proteintech, Wuhan, China). After incubation with appropriate horseradish peroxidase (HRP)-conjugated secondary antibodies, immunoreactive bands were detected using the SuperSignal^TM^ West Pico Plus chemiluminescent substrate (34580, Thermo Fisher Scientific). Band intensities were quantified by densitometric analysis using ImageJ software.

### Proteomic analysis of exosomes

After exosome protein extraction, samples were subjected to enzymatic digestion, desalting, and C18 column cleanup, followed by liquid chromatography-tandem mass spectrometry (LC-MS/MS) analysis on a Q Exactive^TM^ HF-X system (Thermo Fisher Scientific) using data-independent acquisition (DIA) mode. Raw data were processed using IA-neural network (NN) software (version 1.8.1) and searched against the UniProt human protein database. Quantitative results were normalized, and differential expression analysis was performed using the limma package in R. Screening criteria were set as |log_2_(FC)| ≥ 1.00 (where FC = fold change) and *P* ≤ 0.01 (reducing the risk of false positives and ensuring that the selected proteins undergo significant and reliable changes under experimental conditions, thereby increasing data credibility, as supported by relevant literature^[25-27]^). Functional annotation of differentially expressed proteins (DEPs) was performed as follows. Gene Ontology (GO) enrichment analysis (http://www.geneontology.org/) was conducted using the ClusterProfiler package in R, based on a hypergeometric distribution algorithm to test statistical significance. *P* values were corrected for multiple testing using the false discovery rate (FDR), and *Q* values were calculated. Kyoto Encyclopedia of Genes and Genomes (KEGG) enrichment analysis (http://www.kegg.jp/) was conducted using the ClusterProfiler package in R with an over-representation analysis (ORA) method. Significance testing was based on a hypergeometric distribution, with *P* values corrected by FDR and *Q* values calculated.

### miRNA microarray detection and analysis of exosomes

Total RNA isolated from exosomes was first subjected to quality assessment, and samples meeting the quality criteria were subsequently analyzed using a miRNA microarray platform (miRNA-4.0). The experimental procedure comprised RNA isolation (Exosome RNA Purification Kit, 5202050-50T, SIMGEN, Hangzhou, China), RNA quantification and integrity evaluation (Qubit^TM^ microRNA Assay Kit, Q32881, Thermo Fisher Scientific), reverse transcription (PrimeScript^TM^ RT Reagent Kit, RR037A, Takara, Shiga, Japan), and quantitative PCR (qPCR) assessment [Premix Ex Taq^TM^ (Probe qPCR), RR390A, Takara]. For labeling, total RNA was processed by poly(A) tailing according to the manufacturer’s instructions for the Affymetrix FlashTag kit, including the addition of RNA Spike Control Oligos and Poly(A) Tailing Master Mix, followed by incubation at 37 °C for 15 min. Subsequent biotin labeling was performed using 5× FlashTag Biotin HSR Ligation Mix together with T4 DNA Ligase. Labeled RNA samples were then hybridized onto the appropriate microarray chips in a GeneChip Hybridization Oven 645 (Affymetrix, USA) under controlled temperature and rotation conditions. Post-hybridization processing, including washing and staining, was carried out using a GeneChip Fluidics Station 450, followed by signal acquisition with a GeneChip 3000 7G scanner. Raw fluorescence data were converted into probe-level signal intensities using GeneChip Operating System (GCOS) software, generating CEL files for downstream analysis. Data processing and bioinformatic analyses were performed using Transcriptome Analysis Console (TAC) software (version 4.0.1) with default Affymetrix analytical settings. Signal intensities were normalized using the Robust Multichip Analysis (RMA) algorithm combined with global scaling. Differentially expressed genes (DEGs) were identified using the limma package in R (version 3.36.5), with a significance threshold of *P* < 0.05 and a FC > 2. GO enrichment analysis was conducted using a two-tailed Fisher’s exact test, with multiple comparisons adjusted by the Benjamini-Hochberg procedure to obtain FDR-corrected *P* values^[28]^. Terms with adjusted *P* < 0.01 were considered statistically significant. KEGG pathway enrichment analysis was performed using the same statistical approach, with significance defined as an adjusted *P* < 0.05.

### Data collection

Proteomic datasets derived from exosomes were analyzed using DIA-NN (version 1.8.1; https://github.com/vdemichev/DiaNN) with the Homo sapiens SWISS-PROT (SP) database (81,837 proteins; UniProt, UP000005640). miRNA 4.0 microarray datasets were generated using the Affymetrix GeneChip platform, and all data were normalized prior to subsequent analyses.

### Statistical analysis

Experimental data are expressed as the mean value with corresponding standard deviation (SD), derived from three independent biological replicates. All statistical calculations and figure generation were carried out using GraphPad Prism version 9. For comparisons involving two groups, parametric Student’s *t* test was applied when data followed a normal distribution, Welch’s *t* test was used in cases of unequal variance, and the Mann-Whitney *U* test was employed for nonparametric data. For analyses involving more than two groups, one-way analysis of variance (ANOVA) was performed, followed by Tukey’s post hoc multiple comparison test. Statistical significance was defined as *P* < 0.05.

## RESULTS

### APS-Lat-EXO promotes latent HIV-1 reactivation in J-Lat 10.6 cells

**Figure 1:**
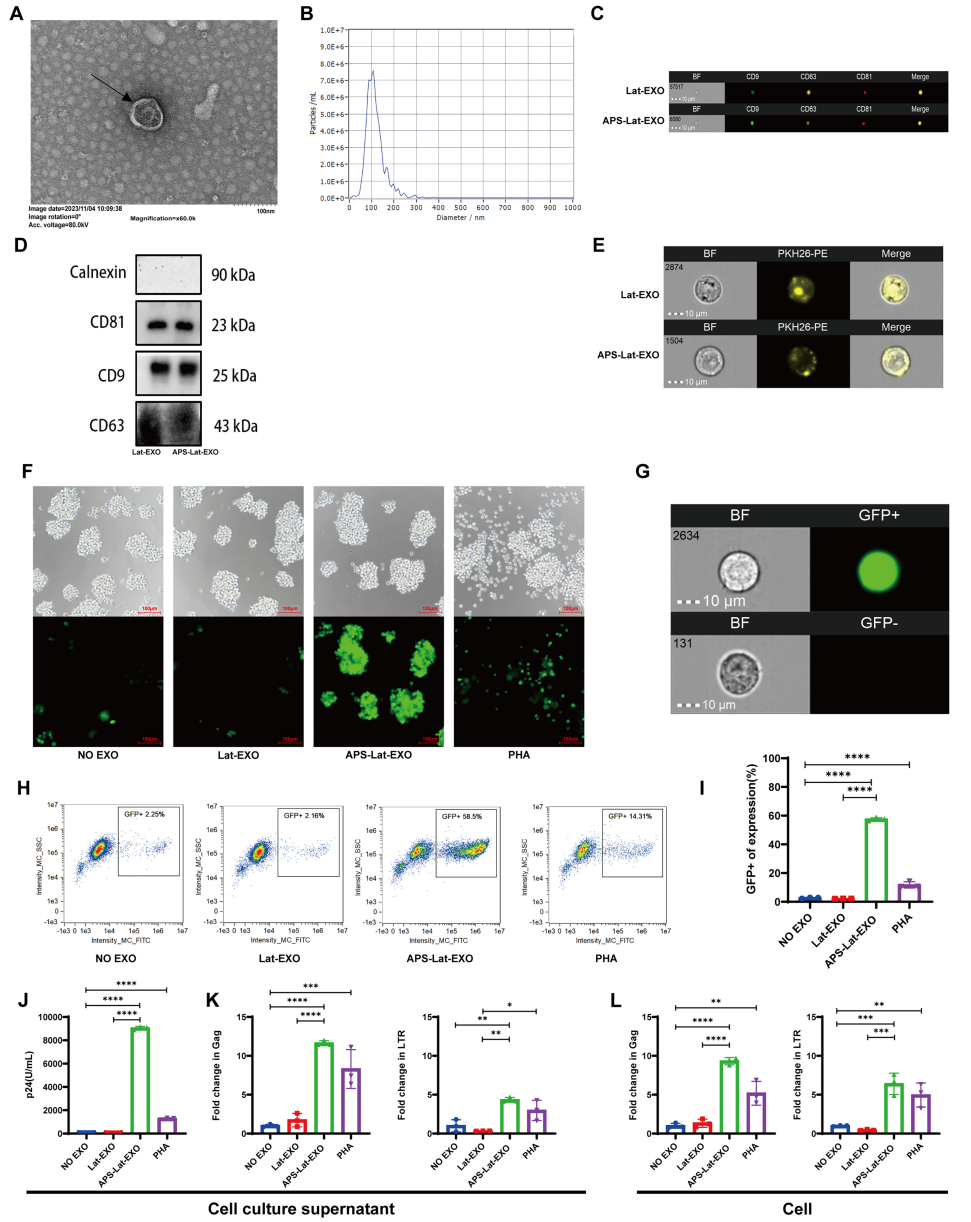
Characterization of APS-Lat-EXO and its ability to induce latent HIV-1 reactivation in J-Lat 10.6 cells. (A) Representative transmission electron microscopy images showing the morphology of isolated exosomes (scale bar, 100 nm); (B) Nanoparticle tracking analysis demonstrating the size distribution of APS-Lat-EXO, with a major peak around 105 nm; (C) Detection of exosomal surface markers (CD9-FITC, CD63-PE, CD81-APC) by Amnis imaging flow cytometry; (D) Expression of exosomal marker proteins (CD9, CD63, and CD81) confirmed by Western blotting; (E) Uptake of PKH-26-labeled exosomes by J-Lat 10.6 cells, visualized using Amnis imaging flow cytometry; (F) Representative fluorescence microscopy images (20×) showing GFP expression in J-Lat 10.6 cells following treatment; (G and H) Flow cytometric quantification of GFP-positive cell populations, with boxed areas indicating GFP⁺ cells; (I) Summary analysis of the proportion of GFP-expressing cells; (J) HIV-1 p24 levels in culture supernatants measured by ELISA (100-fold dilution); (K and L) Relative expression levels of *Gag* and *LTR* transcripts in cells and culture supernatants assessed by RT-qPCR. APS was applied at 800 μg/mL, while APS-Lat-EXO and Lat-EXO were used at 15 μg/mL. PHA (positive control) was used at 5 μg/mL. Data are presented as mean ± SD from three independent experiments (*n* = 3). Statistical significance was determined using Tukey’s multiple comparisons test. ^*^P < 0.05; ^**^P < 0.01; ^***^P < 0.001; ^****^P < 0.0001. APS: Astragalus polysaccharide; EXO: exosome; APS-Lat-EXO: APS-modified latency-reversing exosome; HIV-1: human immunodeficiency virus type 1; J-Lat: Jurkat latency model; TEM: transmission electron microscopy; NTA: nanoparticle tracking analysis; FITC: fluorescein isothiocyanate; PE: phycoerythrin; APC: allophycocyanin; Amnis IFC: Amnis imaging flow cytometry; GFP: green fluorescent protein; ELISA: enzyme-linked immunosorbent assay; RT-qPCR: reverse transcription quantitative polymerase chain reaction; PHA: phytohemagglutinin; SD: standard deviation; CD9: cluster of differentiation 9; CD63: cluster of differentiation 63; CD81: cluster of differentiation 81; PKH26: Paul Karl Horan 26; p24: HIV-1 capsid protein p24; Gag: group-specific antigen; LTR: long terminal repeat; BF: bright field.

APS-Lat-EXO were characterized by transmission electron microscopy (TEM; Figure 1A) and nanoparticle tracking analysis (NTA; Figure 1B). The vesicles exhibited a mean diameter of approximately 115.2 nm, which is within the typical size range of exosomes (30-150 nm)^[29]^. Western blot analysis further confirmed the expression of canonical exosomal markers, including CD9, CD63, and CD81 [Figure 1C and D]. In addition, exosomes labeled with PKH26 (Paul Karl Horan 26) fluorescent dye demonstrated effective binding to J-Lat 10.6 cells [Figure 1E].

To determine the effect of APS-Lat-EXO on the HIV-1 latent reservoir, we examined viral reactivation. APS-Lat-EXO treatment significantly increased the proportion of GFP-positive J-Lat 10.6 cells compared with Lat-EXO and untreated controls (Figure 1F-I, *P* < 0.0001). Consistently, HIV-1 p24 levels in culture supernatants were markedly elevated in the APS-Lat-EXO group [Figure 1J]. Further RT-qPCR analysis demonstrated significant increases in both intracellular and extracellular transcripts of group-specific antigen (Gag) and long terminal repeat (LTR) [Figure 1K and L].

Collectively, these results indicate that APS-Lat-EXO effectively induces transcriptional reactivation of latent HIV-1 and promotes viral particle release in J-Lat 10.6 cells.

### Proteins enriched in APS-Lat-EXO are associated with latent HIV-1 reactivation and the PI3K/AKT signaling pathway

**Figure 2:**
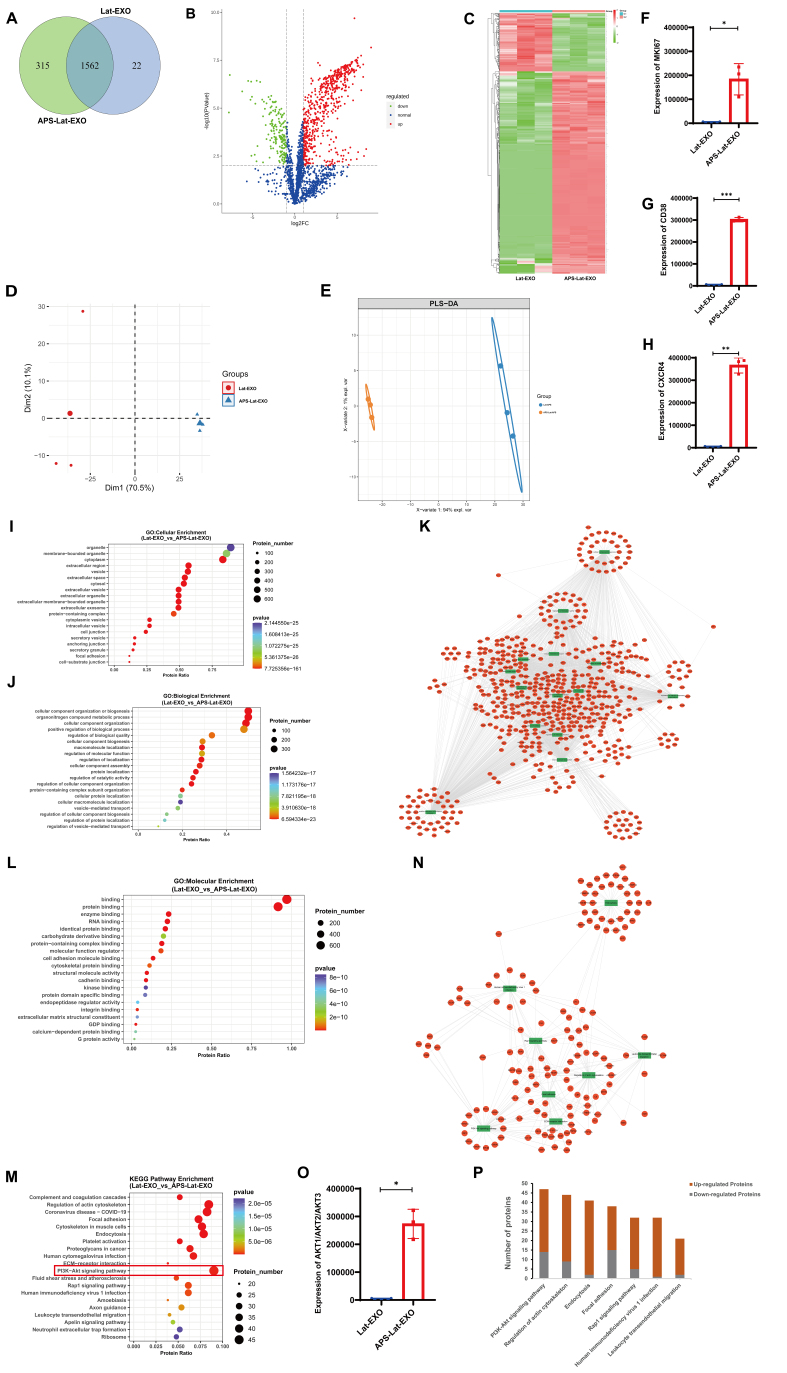
Differential protein expression and functional analyses of APS-Lat-EXO and Lat-EXO. (A) Venn diagram showing uniquely and commonly identified proteins in APS-Lat-EXO and Lat-EXO; (B) Volcano plot of differentially expressed proteins, with FC shown on the log_2_ scale (x-axis) and statistical significance expressed as -log_10_ *P* values (y-axis). Proteins with increased abundance are highlighted in red, while those with decreased abundance are shown in green (|log_2_FC| ≥ 1.00; *P* ≤ 0.01); (C) Hierarchical clustering heatmap of protein expression profiles in APS-Lat-EXO and Lat-EXO, with three biological replicates per group; (D) PCA score plot showing the distribution of samples along the first two principal components; (E) PLS-DA score plot based on 758 differentially expressed proteins screened using Ranking-PCA; (F-H) Expression levels of MKI67, CD38, and CXCR4 in exosomes; (I-K) GO enrichment analysis of the 758 differentially expressed proteins, showing the top 20 categories in CC, biological process, and molecular function; (L) KEGG pathway enrichment analysis of the same protein set, showing the top 20 significantly enriched pathways; (M) Representative subsets of differentially expressed proteins associated with PI3K/AKT signaling, actin cytoskeleton regulation, endocytosis, focal adhesion, Rap1 signaling, HIV-1 infection, and leukocyte transendothelial migration; (N) Expression levels of AKT1, AKT2, and AKT3 in exosomes; (O) Cytoscape network map of enriched GO terms; (P) Cytoscape network map of enriched KEGG pathways. Statistical analysis was performed using an unpaired *t* test with Welch’s correction (*n* = 3). Data are presented as mean ± SD. ^*^P < 0.05; ^**^P < 0.01; ^***^P < 0.001. APS: Astragalus polysaccharide; APS-Lat-EXO: APS-modified latency-reversing exosome; log_2_FC: log_2_ fold change; PCA: principal component analysis; PLS-DA: partial least squares discriminant analysis; MKI67: marker of proliferation Ki-67; CD38: cluster of differentiation 38; CXCR4: C-X-C chemokine receptor type 4; GO: Gene Ontology; KEGG: Kyoto Encyclopedia of Genes and Genomes; PI3K: phosphoinositide 3-kinase; AKT: protein kinase B; SD: standard deviation.

Proteomic analysis of Lat-EXO and APS-Lat-EXO was performed using DIA MS. A higher number of proteins was identified in APS-Lat-EXO than in Lat-EXO (1,877 *vs*. 1,584; Figure 2A; Supplementary Tables 2-4). Differential expression analysis using the limma package (*P* ≤ 0.01) revealed 588 upregulated and 170 downregulated proteins in APS-Lat-EXO [Figure 2B and Supplementary Table 5]. Hierarchical clustering analysis showed a clear separation of the overall protein expression profiles between APS-Lat-EXO and Lat-EXO [Figure 2C]. Consistently, principal component analysis (PCA) demonstrated distinct separation along the first principal component (Dim1), which explained 70.5% of the total variance [Figure 2D]. Partial least squares discriminant analysis (PLS-DA) further confirmed this separation along the first component (X-variate 1), accounting for 94% of the total variance [Figure 2E], indicating substantial differences in protein expression profiles between the two exosome types. Notably, several proteins closely associated with HIV-1 expression, including MKI67 (marker of proliferation Ki-67), CD38, and CXCR4 (C-X-C motif chemokine receptor 4), were significantly upregulated in APS-Lat-EXO (Figure 2F-H; *P* < 0.05; Supplementary Table 5)^[23,30-32]^, suggesting a potential role of the APS-Lat-EXO protein composition in promoting latent HIV-1 reactivation.

GO annotation of DEPs showed that the top 20 enriched Cellular Component (CC) terms were predominantly associated with vesicles, extracellular vesicles, and extracellular exosomes [Figure 2I]. In the Biological Process (BP) category, enriched terms were mainly related to CC organization or biogenesis, protein localization, vesicle-mediated transport, and positive regulation of BP [Figure 2J and K]. In the Molecular Function (MF) category, enrichment was primarily observed in binding-related functions, including protein binding, enzyme binding, and RNA binding [Figure 2L]. Collectively, these findings indicate that APS-Lat-EXO proteins not only exhibit canonical exosomal characteristics but also participate in diverse molecular recognition and regulatory processes that may contribute to the reactivation of latent HIV-1.

Analysis of KEGG pathway enrichment showed that the DEPs were involved in 137 significantly enriched pathways (adjusted *P* < 0.05; Supplementary Table 6). Among the top-ranked enriched pathways were PI3K/AKT signaling, actin cytoskeleton regulation, endocytosis, focal adhesion, Ras-associated protein-1 (Rap1) signaling, HIV-1 infection, and leukocyte transendothelial migration [Figure 2M and N]. These pathways are closely associated with T-cell activation and HIV-1-related processes, contributing to viral reactivation and immune responses through coordinated regulation of cytoskeletal dynamics, cell adhesion and migration, endocytic trafficking, and viral membrane fusion. Notably, the PI3K/AKT signaling pathway contained the highest number of enriched DEPs, with AKT family members (AKT1, AKT2, AKT3) significantly upregulated in APS-Lat-EXO [Figure 2O]. Moreover, most of the proteins enriched in these pathways showed an upward trend [Figure 2P]. These findings suggest that APS-Lat-EXO may enhance PI3K/AKT signaling activity via upregulated proteins, thereby promoting the activation of latent HIV-1.

### Differential miRNAs in APS-Lat-EXO are associated with transcription factors regulating latent HIV-1 activation

**Figure 3:**
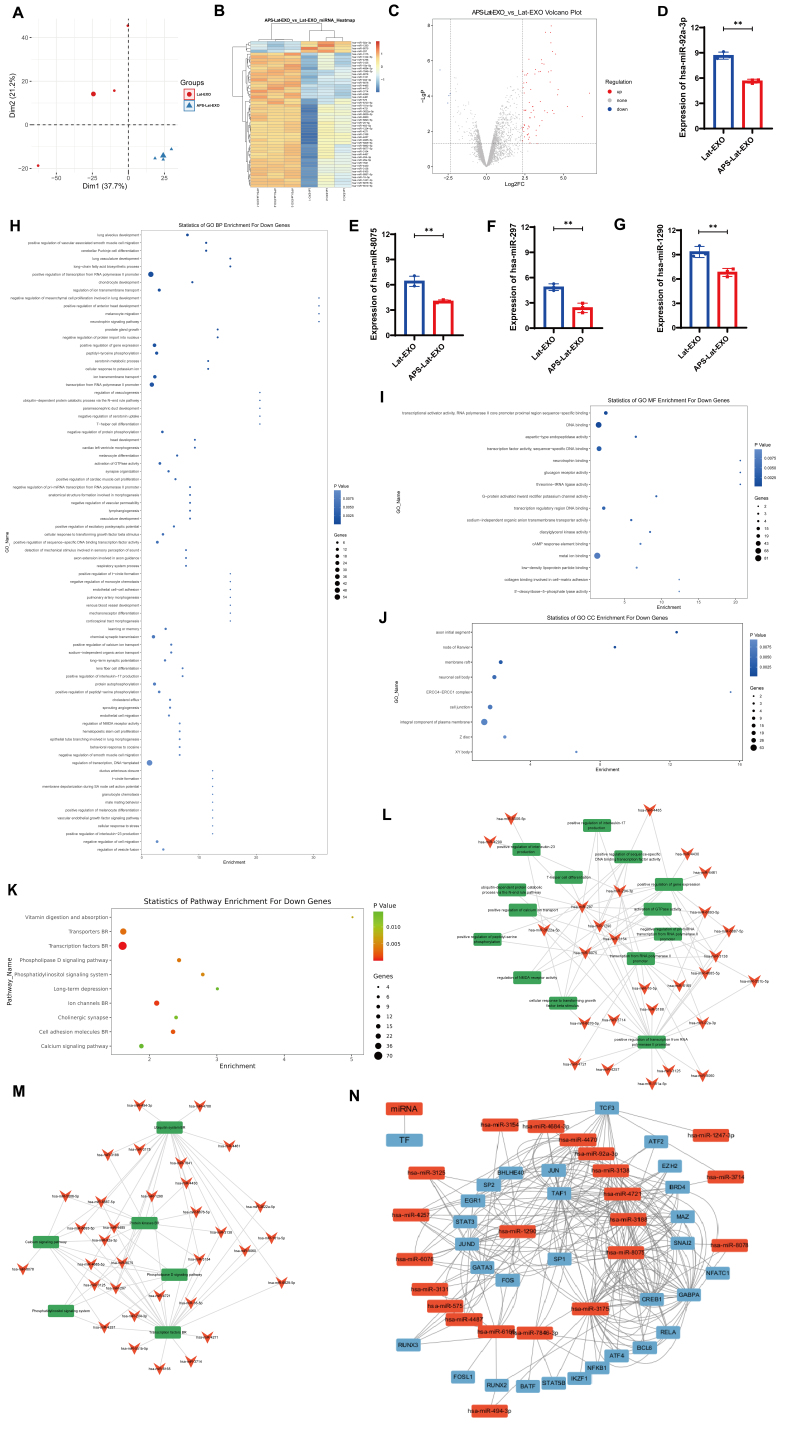
Differential miRNA expression and functional analyses of APS-Lat-EXO and Lat-EXO. (A) PCA plot based on exosomal miRNA expression profiles; (B) Hierarchical clustering heatmap of differentially expressed miRNAs, with three biological replicates per group; (C) Volcano plot depicting miRNA expression changes between APS-Lat-EXO and Lat-EXO; log_2_ fold change is plotted on the horizontal axis and -log_10_ *P* value on the vertical axis. Upregulated miRNAs are highlighted in red, whereas downregulated miRNAs are shown in blue (*P* < 0.05, FC > 5); (D-G) Relative expression levels of the four miRNAs that were downregulated (hsa-miR-92a-3p, hsa-miR-8075, hsa-miR-297, and hsa-miR-1290); (H-J) Gene Ontology enrichment results of predicted target genes associated with the four downregulated miRNAs; (K) KEGG pathway enrichment analysis of the corresponding target genes; (L) Cytoscape-based network visualization of miRNA target genes annotated by GO terms; (M) Cytoscape network representation of miRNA target genes mapped to KEGG pathways; (N) Cytoscape network map of predicted TF-miRNA interactions. Statistical comparisons were performed using an unpaired *t* test with Welch’s correction (*n* = 3). Data are presented as mean ± SD. ^**^P < 0.01. APS: Astragalus polysaccharide; APS-Lat-EXO: APS-modified latency-reversing exosome; PCA: principal component analysis; FC: fold change; GO: Gene Ontology; KEGG: Kyoto Encyclopedia of Genes and Genomes; TF: transcription factor; SD: standard deviation.

Exosomes carry diverse classes of nucleic acids, such as RNA, DNA, and miRNAs, and their molecular composition largely mirrors that of the cells from which they originate^[33]^. To compare the miRNA expression profiles of Lat-EXO and APS-Lat-EXO, we performed miRNA 4.0 microarray analysis. PCA demonstrated a clear separation of the two groups based on global miRNA expression profiles [Figure 3A], and hierarchical clustering analysis (HCA) confirmed this trend [Figure 3B]. Fifty-one miRNAs were found to be differentially expressed in APS-Lat-EXO (*P* < 0.05, FC > 5), comprising 47 upregulated and 4 downregulated miRNAs [Figure 3C and Supplementary Table 7]. Among these were hsa-miR-92a-3p, hsa-miR-8075, hsa-miR-297, and hsa-miR-1290 [Figure 3D-G]. Previous studies have reported that exosomal overexpression of hsa-miR-92a-3p suppresses PI3K/AKT signaling via targeting RAP1B, thereby affecting glioma cell migration and invasion^[34]^; miR-297 overexpression inhibits HCC cell proliferation by downregulating PTBP3 and suppressing the PI3K/AKT pathway^[35]^; elevated miR-1290 reduces p-AKT and downstream NF-κB-related proteins^[36]^; while miR-8075 is downregulated in cervical cancer tissues, and its inhibition upregulates TPX2, promoting cell proliferation and migration^[37]^. These results indicate that differentially expressed miRNAs in APS-Lat-EXO may influence target cell proliferation and signaling through multiple mechanisms.

To investigate the biological relevance of the differentially expressed miRNAs, potential target genes were predicted using MiRanda and TargetScan, and the overlapping targets were retained for subsequent analysis [Supplementary Table 8]. GO and KEGG enrichment analyses were subsequently conducted (*P* < 0.01). In GO annotation, 79 BP terms were identified, with enrichment mainly in “positive regulation” processes (13 terms), including positive regulation of vascular-associated smooth muscle cell migration, positive regulation of transcription from RNA polymerase II promoter, and positive regulation of gene expression [Figure 3H and Supplementary Table 9]. In MF, 16 significantly enriched terms were identified, primarily involving binding activities (8 terms), such as RNA polymerase II core promoter proximal region sequence-specific binding, DNA binding, and sequence-specific DNA binding [Figure 3I and Supplementary Table 10]. In CC, nine significantly enriched terms were associated with membrane structures, including integral component of plasma membrane, cell junction, and membrane raft [Figure 3J and Supplementary Table 11]. These results indicate that the differentially enriched miRNAs present in APS-Lat-EXO are likely involved in modulating transcriptional processes within recipient cells. KEGG pathway enrichment analysis revealed the top 10 significantly enriched pathways (adjusted *P* < 0.05), including the Phospholipase D signaling pathway, Phosphatidylinositol signaling system, and Calcium signaling pathway [Figure 3K and Supplementary Table 12]. These pathways are closely related to T-cell activation, suggesting that APS-Lat-EXO may participate in the regulation of T-cell functions.

A regulatory network constructed based on the differentially expressed miRNAs further demonstrated that hsa-miR-92a-3p, hsa-miR-8075, hsa-miR-297, and hsa-miR-1290 were involved in multiple critical BPs and signaling pathways [Figure 3L and M]. To investigate upstream mechanisms, we predicted transcription factor (TF)-miRNA interactions. The results indicated that the activator protein 1 (AP-1) family (JUN, JUND, FOS), NF-κB family (NFKB1), and runt‑related transcription factor (RUNX) family (RUNX2, RUNX3) may commonly regulate several key miRNAs [Figure 3N]. Notably, the HIV-1 5’LTR promoter region is rich in binding sites for these TFs, and previous studies have reported that they positively regulate HIV-1 transcription^[38,39]^, suggesting that APS-Lat-EXO may be enriched in transcriptional regulators related to latent HIV-1 activation. In addition, TFs such as GATA binding protein 3 (GATA3), activating transcription factor (ATF) family members, specificity protein 1 (SP1), and early growth response 1 (EGR1) showed potential regulatory relationships with multiple differentially expressed miRNAs. Among them, GATA3 expression is positively correlated with the level of latent HIV-1 activation^[30]^; ATF and SP1 likewise play potential roles in HIV-1 transcriptional activation^[30]^; and EGR1 has been reported to be closely associated with HIV-1 activation in primary CD4+ T cells from infected individuals^[30]^.

Collectively, these findings indicate that miRNAs differentially enriched in APS-Lat-EXO are not only closely linked to TFs involved in latent HIV-1 activation, but may also participate in the PI3K/AKT signaling pathway, thereby providing important theoretical insights and directions for elucidating the mechanisms underlying HIV-1 latency reversal.

### APS-Lat-EXO activates latent HIV-1 via the PI3K/AKT/NF-κB signaling pathway

**Figure 4:**
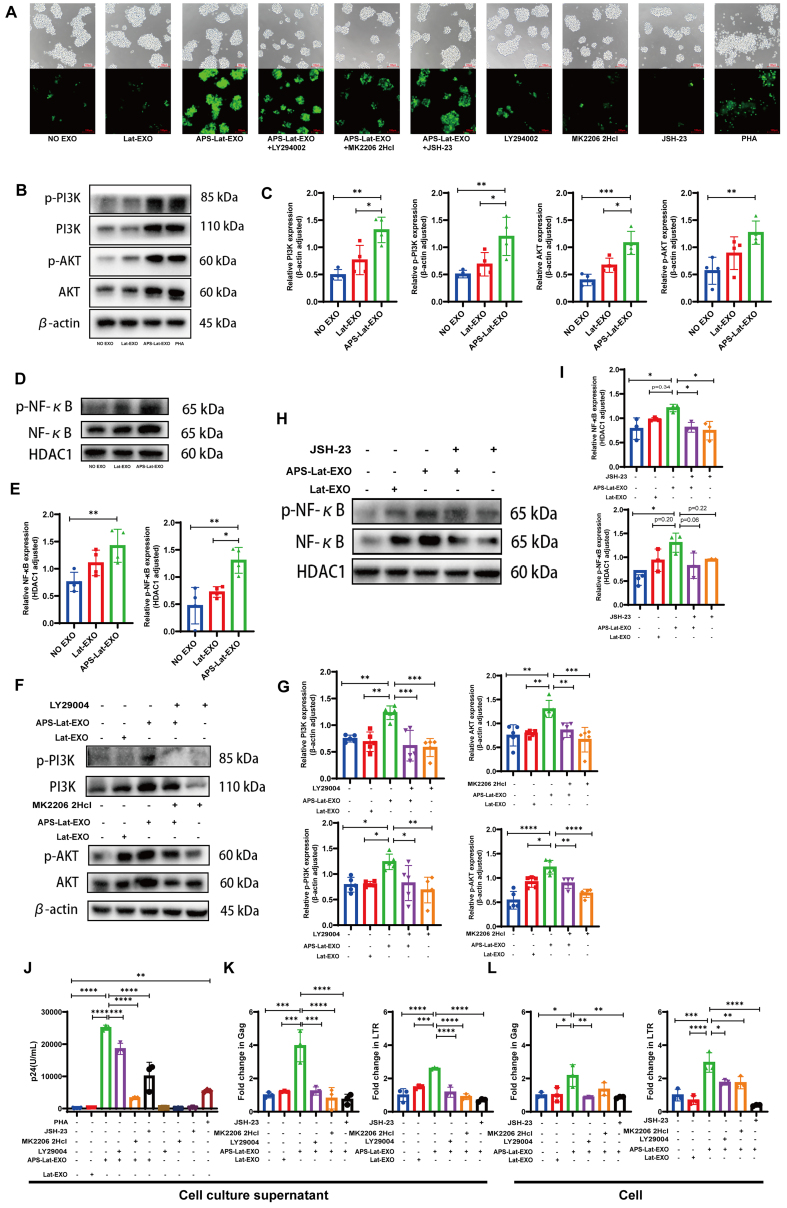
APS-Lat-EXO reactivates HIV-1 provirus through the PI3K/AKT/NF-κB signaling pathway. (A) Representative fluorescence microscopy images showing GFP expression across different treatment conditions (20×). Cells were exposed to LY294002 (5 μM), MK2206 2HCl (0.5 μM), or JSH-23 (1 μM) as pathway inhibitors; (B, D, F and H) Immunoblot analysis of PI3K, phosphorylated PI3K, AKT, phosphorylated AKT, NF-κB, and phosphorylated NF-κB following 48 h exposure to APS-Lat-EXO, either alone or together with the indicated inhibitors; (C, E, G and I) Densitometric analysis corresponding to the immunoblot results; (J) Quantification of HIV-1 p24 levels in culture supernatants by ELISA (100× dilution); (K and L) RT-qPCR measurement of Gag and LTR transcript abundance in both supernatants and cellular fractions. Results are derived from at least three independent experiments, with individual data points representing separate samples. Statistical differences were determined using Tukey’s multiple comparisons test (C/E, *n* = 4; G, *n* = 5; I/J/K/L, *n* = 3). Data are presented as mean ± SD. ^*^P < 0.05; ^**^P < 0.01; ^***^P < 0.001; ^****^P < 0.0001. APS: Astragalus polysaccharide; EXO: exosome; Lat-EXO: latency-reversing exosome; APS-Lat-EXO: APS-modified latency-reversing exosome; PI3K: phosphoinositide 3-kinase; AKT: protein kinase B; NF-κB: nuclear factor kappa-light-chain-enhancer of activated B cells; GFP: green fluorescent protein; LY294002: PI3K inhibitor; MK2206 2HCl: AKT inhibitor; JSH-23: NF-κB inhibitor; p24: HIV-1 capsid protein p24; ELISA: enzyme-linked immunosorbent assay; RT-qPCR: reverse transcription quantitative polymerase chain reaction; SD: standard deviation; Gag: group-specific antigen; LTR: long terminal repeat.

To assess how APS-Lat-EXO influences the PI3K/AKT/NF-κB signaling pathway, J-Lat 10.6 cells were treated in combination with LY294002 (a PI3K inhibitor), MK2206 2HCl (an AKT inhibitor), and JSH-23 (an NF-κB inhibitor). Fluorescence microscopy revealed that APS-Lat-EXO treatment markedly enhanced green fluorescence intensity in the cells, whereas the addition of inhibitors attenuated the fluorescence signal [Figure 4A]. Consistent with this, Western blot analysis demonstrated that APS-Lat-EXO significantly increased the expression of PI3K, p-PI3K, AKT, p-AKT, and nuclear proteins NF-κB and phospho-nuclear factor kappa B (p-NF-κB) [Figure 4B-E], indicating activation of the PI3K/AKT/NF-κB pathway. Co-treatment with LY294002, MK2206 2HCl, or JSH-23 led to a decrease in the expression of these proteins [Figure 4F-I]. ELISA further showed that p24 levels in the culture supernatant were significantly reduced in the inhibitor combination groups compared with APS-Lat-EXO treatment alone [Figure 4J]. RT-qPCR analysis confirmed that co-treatment with inhibitors markedly downregulated both intracellular and extracellular levels of Gag and LTR transcripts [Figure 4K and L]. In addition, flow cytometry revealed a significant reduction in the proportion of GFP⁺ cells [Supplementary Figure 1].

Taken together, these results demonstrate that APS-Lat-EXO effectively reactivates latent HIV-1 by activating the PI3K/AKT/NF-κB signaling pathway, highlighting its potential as a latency reversal strategy.

### APS-Lat-EXO induces reactivation of latent HIV-1 in PBMCs from HIV-infected individuals

**Figure 5:**
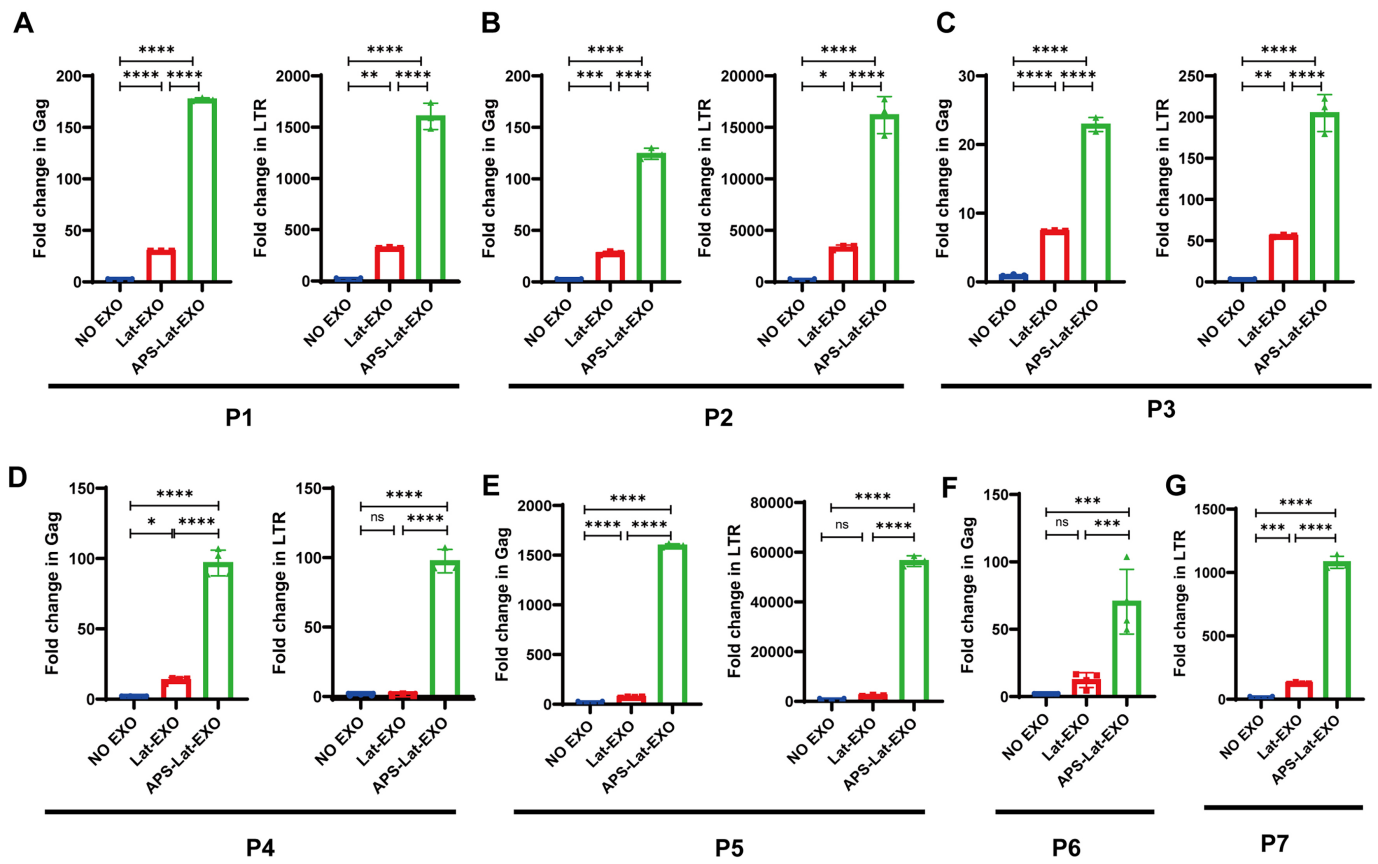
APS-Lat-EXO reactivates latent HIV-1 in PBMCs from ART-treated patients(*n* = 7). (A-G) RT-qPCR assessment of Gag and LTR transcript abundance in PBMCs derived from three ART-suppressed HIV-1-infected donors (P1, P2, P3). Each data point corresponds to an individual donor sample. Statistical differences were determined using Tukey’s multiple comparisons test. Data are presented as mean ± SD. ^*^P < 0.05; ^**^P < 0.01; ^***^P < 0.001; ^****^P < 0.0001. APS: Astragalus polysaccharide; APS-Lat-EXO: APS-modified latency-reversing exosome; PBMC: peripheral blood mononuclear cell; ART: antiretroviral therapy; RT-qPCR: reverse transcription quantitative polymerase chain reaction; Gag: group-specific antigen; LTR: long terminal repeat; SD: standard deviation.

To further assess the capacity of APS-Lat-EXO to induce HIV-1 reactivation in primary cells, peripheral blood mononuclear cells (PBMCs) isolated from individuals receiving ART were incubated with APS-Lat-EXO or Lat-EXO. The relevant clinical characteristics of the seven participants are shown in Table 1. RT-qPCR was used to measure the relative expression of HIV-1 Gag and LTR transcripts. The results showed that APS-Lat-EXO treatment significantly increased Gag and LTR transcript levels in PBMCs from all three patients tested [Figure 5A-G]. These findings indicate that APS-Lat-EXO can induce HIV-1 RNA expression in patient-derived cells, supporting its potential application as a biologically derived LRA.

**Table 1:** The clinical test information of the seven participants

**Patient ID**	**Sex**	**ART**	**CD4 count/µL**	**CD8 count/µL**	**CD4+T/CD8+T** **(0.71-2.78)**	**Viral load (copies/mL)**	**ALT (9-50)**	**AST** **(15-40)**
P1	M	Treated	328	1,071	0.31	<40	18	19
P2	M	Treated	690	1,192	0.58	TND	45	32
P3	M	Treated	603	509	1.18	TND	14	17
P4	M	Treated	713	617	1.16	TND	21	17
P5	M	Treated	689	464	1.48	TND	42	26
P6	M	Treated	846	1051	0.8	TND	43	40
P7	M	Treated	775	633	1.22	TND	18	20

ART: Antiretroviral therapy; ALT: alanine aminotransferase; AST: aspartate aminotransferase; CD: cluster of differentiation; M: male; TND: Target Not Detected; LLOD: Lower Limit of Detection (40 copies/mL); Treated: HIV patients on ART.

## DISCUSSION

APS, a class of highly bioactive natural polysaccharides extracted from the traditional Chinese medicinal herb *Astragalus membranaceus*, have been increasingly investigated in recent years for their adjunctive roles in viral diseases, tumor immunology, and autoimmune disorders^[40,41]^. Previous studies have demonstrated that APS can engage the Toll-like receptor 4 (TLR4)-Myeloid differentiation primary response 88 (MYD88) signaling cascade and activate downstream transcriptional regulators such as NF-κB and AP-1, thereby contributing to its immunostimulatory and antitumor activities^[42]^. In addition, APS can promote T-cell differentiation, suggesting that it positively regulates the initial activation of T cells^[43]^. Within HIV-1 latency reversal strategies, the activation of resting CD4⁺ T cells can induce the expression of latent virus^[44]^. Therefore, the ability of APS to enhance T-cell immune functions through activation of specific signaling pathways provides a theoretical basis for its potential role as a LRA within the “shock and kill” strategy.

Exosomes, owing to the heterogeneity of their protein, nucleic acid, and lipid components, exert diverse biological effects in immune regulation, either promoting or suppressing T-cell activation depending on the characteristics of their cells of origin^[45]^. Accumulating evidence indicates that exosomes released from HIV-1-infected cells carry viral accessory proteins and pro-inflammatory mediators, which are involved in promoting HIV-1 activation, replication, and immunopathogenic processes^[46]^. However, strategies that exploit exosomes and their bioactive cargo following drug treatment to reactivate latent HIV-1 remain largely unexplored. In this study, we observed that APS-Lat-EXO could reactivate latent HIV-1 in CD4⁺ T cells. When APS-Lat-EXO was co-cultured with latently infected J-Lat 10.6 cells, viral reactivation occurred, as indicated by a marked increase in GFP expression. This finding suggests that APS-Lat-EXO can interact with J-Lat 10.6 cells, leading to the activation of latent HIV-1 infection and the subsequent release of viral particles.

Proteomic analysis further revealed significant alterations in the protein profile of APS-Lat-EXO. We identified elevated expression of proteins associated with CD4⁺ T-cell activation, proliferation, and HIV-1 infection - namely CD38, MKI67, and CXCR4 - in the APS-Lat-EXO group, suggesting that APS activates CD4⁺ T cells, resulting in the secretion of exosomes enriched with these proteins. Previous studies have reported that the expression levels of CD38 and MKI67 positively correlate with HIV-1 viral RNA (vRNA), while high CXCR4 expression may be linked to increased susceptibility to HIV-1 infection^[23,30]^. These DEPs may therefore play key roles in APS-Lat-EXO-mediated reactivation of latent HIV-1. In addition, the DEPs in APS-Lat-EXO were involved in multiple signaling pathways, particularly those related to leukocyte activation, migration, and HIV-1 infection. These pathways included the PI3K/AKT signaling pathway, regulation of the actin cytoskeleton, and HIV-1 infection. The PI3K/AKT pathway is a critical intracellular signaling cascade that regulates numerous BP, including cell survival, cell cycle progression, and cell growth^[47,48]^. Importantly, studies have shown that PI3K/AKT signaling is enhanced in p24⁺ cells from HIV-1-infected individuals, underscoring its biological relevance in HIV-1 infection^[49]^. The upregulation of proteins involved in these pathways suggests their participation in the activation of effector cell-related signaling cascades. Collectively, these findings highlight potential mechanisms through which APS-Lat-EXO reactivates latent HIV-1.

APS not only alters the protein composition of exosomes but also affects their miRNA expression. Given the stability of miRNAs and their ability to regulate gene expression, their role in exosome-mediated intercellular communication has attracted increasing attention in recent years. Following APS treatment, GO enrichment analysis of downregulated miRNAs in APS-Lat-EXO revealed that the regulated miRNAs were involved in multiple transcription-related processes, while pathway analysis indicated significant enrichment of T-cell activation-related pathways. Previous studies have shown that HIV-1 silencing in CD4⁺ T cells involves multiple mechanisms, including reduced levels of positive TFs such as NF-κB and AP-1^[50,51]^. Based on this, we constructed a TF-miRNA regulatory network to identify key TFs, revealing several known regulators that promote HIV-1 transcription, including NF-κB, AP-1, and Sp1^[52]^, as well as GATA3, JUND, and ATF. A recent study identified GATA3 as a novel TF positively correlated with HIV-1 vRNA expression in functional assays^[30]^, consistent with our findings.

We focused on the PI3K/AKT signaling pathway, which was significantly enriched in our proteomic analysis, and the TF NF-κB, which regulates the expression of differentially expressed miRNAs. Under APS-Lat-EXO treatment, the expression and phosphorylation of these key proteins were markedly upregulated, whereas pathway inhibitor treatment suppressed protein expression and attenuated the reactivation of latent HIV-1. These findings indicate that APS-Lat-EXO induces latent HIV-1 reactivation via the PI3K/AKT/NF-κB signaling pathway.

Further investigation revealed that APS-Lat-EXO exhibited a significant reactivation effect on latent reservoirs in ART-treated HIV-1 patient-derived PBMCs. Interestingly, we also observed that Lat-EXO could reactivate latent HIV-1 in patient-derived cells, whereas in cell line models, Lat-EXO did not show a notable reactivation effect. Supporting evidence from previous studies suggests that cell line models and primary human T cells differ fundamentally in their transcriptomic and epigenetic profiles^[53-55]^. In this study, the J-Lat 10.6 cell line was used as a model for latent HIV-1 infection, offering advantages such as a uniform latent state and the ability to directly quantify activation via GFP expression. However, this model has limitations, including differences from natural HIV infection due to its transfection-based construction and the lack of a complete *in vivo* environment, which may introduce biases in assessing the activation effects of APS-Lat-EXO. Given the limited number of patient samples included in this study, further large-scale validation is required, particularly in diverse HIV-1 latency cell line models and primary cells derived from HIV-1-infected individuals. Although APS-Lat-EXO shows great promise in preclinical settings, it is important to note that translating this approach into clinical use will require careful consideration of factors such as dosing, long-term safety, and immune response modulation. Therefore, while the current study lays a foundation for future *in vivo* and clinical research, significant steps remain before APS-Lat-EXO can be translated into clinical practice. Moving forward, integrating *in vitro* cell models with *in vivo* and clinical validation will be essential for gaining deeper insights into the mechanisms of HIV-1 latency reversal and for developing novel LRAs aimed at reactivating and ultimately eradicating latent HIV-1.

## DECLARATIONS

### Availability of data and materials

Raw data and materials are available from the corresponding authors upon reasonable request.

### AI and AI-assisted tools statement

During the preparation of the manuscript, the authors used AI-assisted tools for sentence refinement to improve the clarity and fluency of the text.

### Copyright

© The Author(s) 2026.
