# Supplementary material for: Astragalus polysaccharide promotes latent HIV-1 reactivation via exosome-mediated modulation of the PI3K/AKT/NF-κB axis
Source: Extracell Vesicles Circ Nucl Acids. 2026 Jan 29;7(1):94–113. doi: 10.20517/evcna.2025.148 (PMC13074303; doi:10.20517/evcna.2025.148)
Supplement: Supplementary file 1 [file evcna-7-1-94-SupplementaryMaterials.zip › evcna60148-SupplementaryMaterials/evcna60148-SupplementaryMaterials.pdf]

Supplementary Materials

Astragalus polysaccharide promotes latent HIV-1 reactivation via exosome-mediated modulation of the PI3K/AKT/NF-κB axis

Fang Xu[1,2,3], Shumin Luo[1,2,3], Pengpeng Lu[1,2,3], Yiyue Wang[1,2,3], Guannan Sun[1,2,3], Chuanyun Li[3], Weihua Li[1,2,3]

[1]Beijing Institute of Hepatology, Beijing You An Hospital, Capital Medical University, Beijing 100069, China.

[2]Beijing Institute of Infectious Diseases of Integrated Traditional Chinese and Western Medicine, Beijing You An Hospital, Capital Medical University, Beijing 100069, China.

[3]Beijing You An Hospital, Capital Medical University, Beijing 100069, China

Correspondence to: Prof. Weihua Li, Beijing Institute of Infectious Diseases of Integrated Traditional Chinese and Western Medicine, Beijing You An Hospital, Capital Medical University, Beijing 100069, China. E-mail: liweihua@ccmu.edu.cn; Dr. Chuanyun Li, Beijing You An Hospital, Capital Medical University, Beijing 100069, China. E-mail:lichuany0388@163.com

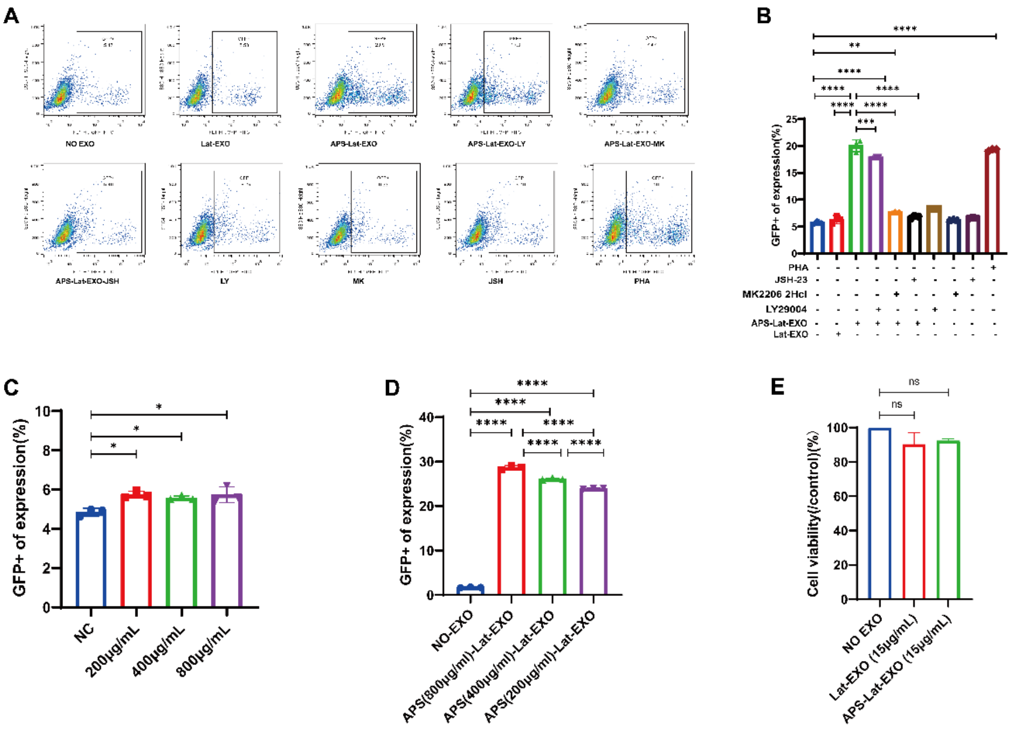

Supplementary Figure 1. (A) Flow cytometric analysis of GFP expression in cells from each treatment group. Inhibitor treatments included LY294002 (5 μM), MK2206 2HCl (0.5 μM), and JSH-23 (1 μM); (B) Statistical analysis of the flow cytometry results; (C) Quantification of GFP expression in cells. Cells were treated with different concentrations of APS, and GFP expression was analyzed by flow cytometry; APS concentrations were 200 μg/mL, 400 μg/mL, and 800 μg/mL; (D) Statistical analysis of flow cytometry results. Cells were treated with different concentrations of APS, and after 48 h, exosomes were collected from the culture supernatants and applied to J-Lat 10.6 cells. GFP expression was measured 48 h later; APS concentrations were 200 μg/mL, 400 μg/mL, and 800 μg/mL; (E) Cell viability assessed by CCK-8 assay. Lat-EXO (15 μg/mL) and APS-Lat-EXO (15 μg/mL) were applied to J-Lat 10.6 cells, and cell viability was measured after 48 h at OD 450 nm.Data are representative of three or more independent experiments, with each data point representing an independent experimental sample. Error bars indicate mean ± SD. Statistical significance was determined using Tukey's multiple comparisons test. *$P < 0.05$; **$P < 0.01$; ***$P < 0.001$; ****$P < 0.0001$. APS: Astragalus polysaccharide; APS-Lat-EXO: APS-modified latency-reversing exosome; GFP: green fluorescent protein; CCK-8: Cell Counting Kit-8; SD: standard deviation.

Supplementary Table 1. Primer sequences of genes

Primer	Sequence(5'to3')
LTR-F	GCCTCCTAGCATTTCGTCACAT
LTR-R	GCTGCTTATATGTAGCATCTGAGG
Gag-F	GTCCAGAATGCGAACCCAGA
Gag-R	GTTACGTGCTGGCTCATTGC
GAPDH-F	CTCTGCTCCTCCTGTTCGAC
GAPDH-R	AGTTAAAAGCAGCCCTGGTGA
